# Identification of an *Xiap*-Like Pseudogene on Mouse Chromosome 7

**DOI:** 10.1371/journal.pone.0008078

**Published:** 2009-11-30

**Authors:** Aneta Kotevski, Wendy D. Cook, David L. Vaux, Bernard A. Callus

**Affiliations:** 1 Department of Biochemistry, La Trobe University, Bundoora, Victoria, Australia; 2 Western Australian Institute of Medical Research and School of Biomolecular, Biomedical and Chemical Sciences, University of Western Australia, Crawley, Western Australia, Australia; University of Texas MD Anderson Cancer Center, United States of America

## Abstract

The most thoroughly characterized mammalian IAP is XIAP/BIRC4, which can inhibit caspases 9, 3 and 7, but may also regulate apoptosis through interactions with other proteins such as Smac/DIABLO, HtrA2/Omi, XAF1, TAK1, cIAP1, and cIAP2.

High throughput sequencing of the mouse genome revealed the existence of a gene resembling *Xiap/Birc4* on mouse chromosome 7. To confirm the existence of this gene, and to determine its functional significance, we performed Southern and Northern blot analysis. This showed the presence of the *Xiap*-like gene in both wild-type and *Xiap* gene knock-out mice, but the corresponding mRNA was not detected in any tissues examined by Northern blot. Analysis of the gene sequence in all three possible reading frames predicts that expression of this gene would not give rise to a full-length protein, but only non-functional truncated polypeptides. Because its nucleotide sequence is 92% identical to *Xiap*, but it has no introns corresponding to those of *Xiap*, we conclude that *Xiap-ps1* is a pseudogene generated by retro-transposition of a spliced *Xiap* message to chromosome 7.

## Introduction

The inhibitor of apoptosis proteins (IAPs) are a family of proteins that bear one or more baculoviral IAP repeat (BIR) domains [1], [2]. The most thoroughly characterized mammalian IAP is XIAP/BIRC4, which can inhibit caspases 9, 3 and 7 [2], [3], but may also regulate apoptosis through interactions with other proteins such as Smac/DIABLO, HtrA2/Omi, XAF1, TAK1, cIAP1, and cIAP2 [4], [5].

Surprisingly, although XIAP is the most potent caspase inhibitor of the IAPs [6], the phenotype of *Xiap* knockout mice is very mild [7] and humans harbouring *XIAP/BIRC4* mutations have immune system defects due to abnormal NK cell function, but are otherwise normal [8].

We were alerted (Anthony Uren pers. comm.) to the presence of a *Xiap*-like sequence found on mouse chromosome 7 during sequencing of the mouse genome (see: http://apr2006.archive.ensembl.org/Mus_musculus/domainview?domainentry=IPR001370). To determine whether this gene was functional, and whether redundancy with *Xiap* might explain the subtle phenotype of *Xiap* KO mice, we analysed the sequence of this putative *Xiap*-like gene, confirmed its existence by Southern blot, and used Northern analysis to determine whether it was expressed.

## Materials and Methods

### Ethics Statement

All mouse work was done according to the requirements of La Trobe University Animal Ethics Committees with ethics approval number: AEC 09-01-B. Animals were sacrificed using CO2 asphyxiation and the appropriate organs harvested.

### Cell Culture

Primary wild-type and *Xiap*−/− MEFs isolated from C57BL/6 mice were grown in FMA medium (Dulbecco's Modified Eagle (DME) medium supplemented with 10% (v/v) foetal bovine serum (Gibco, Melbourne VIC), penicillin G (50 U/ml), streptomycin (50 µg/ml), L-glutamine (2 mM), 270 µM L-asparagine and 50 µM 2-mercaptoethanol) in a humidified atmosphere of 10% CO_2_ at 37°C. Confluent 10 cm plates were used for preparing genomic DNA. Cells were harvested, washed and the cell pellets stored at −80°C until required.

### Tissue Samples

Tissue samples (liver, brain, lung, spleen, heart and intestine) were harvested from wild-type and *Xiap* −/− C57BL/6 mice and were snap frozen in liquid nitrogen and stored at −80°C until required.

### Genomic DNA Preparation

The frozen samples were incubated overnight with shaking at 55°C in 3 ml genomic lysis buffer (100 mM Tris-HCl pH 8, 5 mM EDTA pH 8, 0.5% (w/v) SDS, 200 mM NaCl, 500 µg/ml Proteinase K). Genomic DNA was precipitated using isopropanol and resuspended in TE buffer (10 mM Tris, 1 mM EDTA). Genomic DNA (12 µg) was digested to completion using *Bam*H1, *Eco*R1, *Hin*dIII or *Xba*I endonucleases.

### Southern Blot Analyses

Digested DNA samples were separated by electrophoresis on 1% agarose TAE gels containing 0.2 µg/ml ethidium bromide. Afterwards, the gel was denatured in 1.5 M NaCl/0.5 M NaOH for 45 min and then neutralised in 1 M Tris (pH 7.4)/1.5 M NaCl. Separated DNA was transferred onto Zeta-probe (BioRad) nitrocellulose membrane by overnight capillary transfer and fixed by baking at 80°C for 2 hr. The membrane was pre-hybridised for 30 min at 65°C in Rapid-Hyb buffer (Amersham) followed by hybridisation with ^32^P labelled *mXiap* DNA probe at 65°C for 2 hr in Rapid-Hyb buffer according to the manufacturer's specifications. The membrane was washed twice at 65°C for 30 min in 0.3X SSC. The hybridised probe was detected using a Typhoon phosphoimager (Amersham).

The DNA probe was obtained by digesting *Xiap* cDNA to make a 510 bp fragment identical to bases 524 to 1034 of the cDNA of *Xiap*, a region within exon 2 and encoding amino acids 105 to 275 of the protein. Alignment of the probe to *Xiap-ps1* showed 92% identity, indicating that the probe would be able to hybridise to both genes at high stringency.

### RNA Extraction and Northern Blot Analyses

To isolate RNA, frozen tissue samples were homogenised in Trizol (Invitrogen) and RNA purified according to the manufacturer's instructions. Total RNA (10–15 µg) was separated on 1.2% agarose formaldehyde gels, transferred to Hybond-N (Amersham) membrane by capillary transfer and fixed by baking at 80°C. Membranes were pre-hybridised for 30 min at 65°C in Rapid-Hyb buffer followed by hybridisation with the ^32^P labelled *mXiap* DNA probe at 65°C for 2 hr. The final wash was three times for 20 min at 65°C in 0.3X SSC+0.5% SDS, and the hybridised probe was detected using a Typhoon phosphorimager (Amersham).

## Results

### Identification of a *Xiap*-Like Gene (*Xiap-ps1*) on Chromosome 7

**Figure 1:**
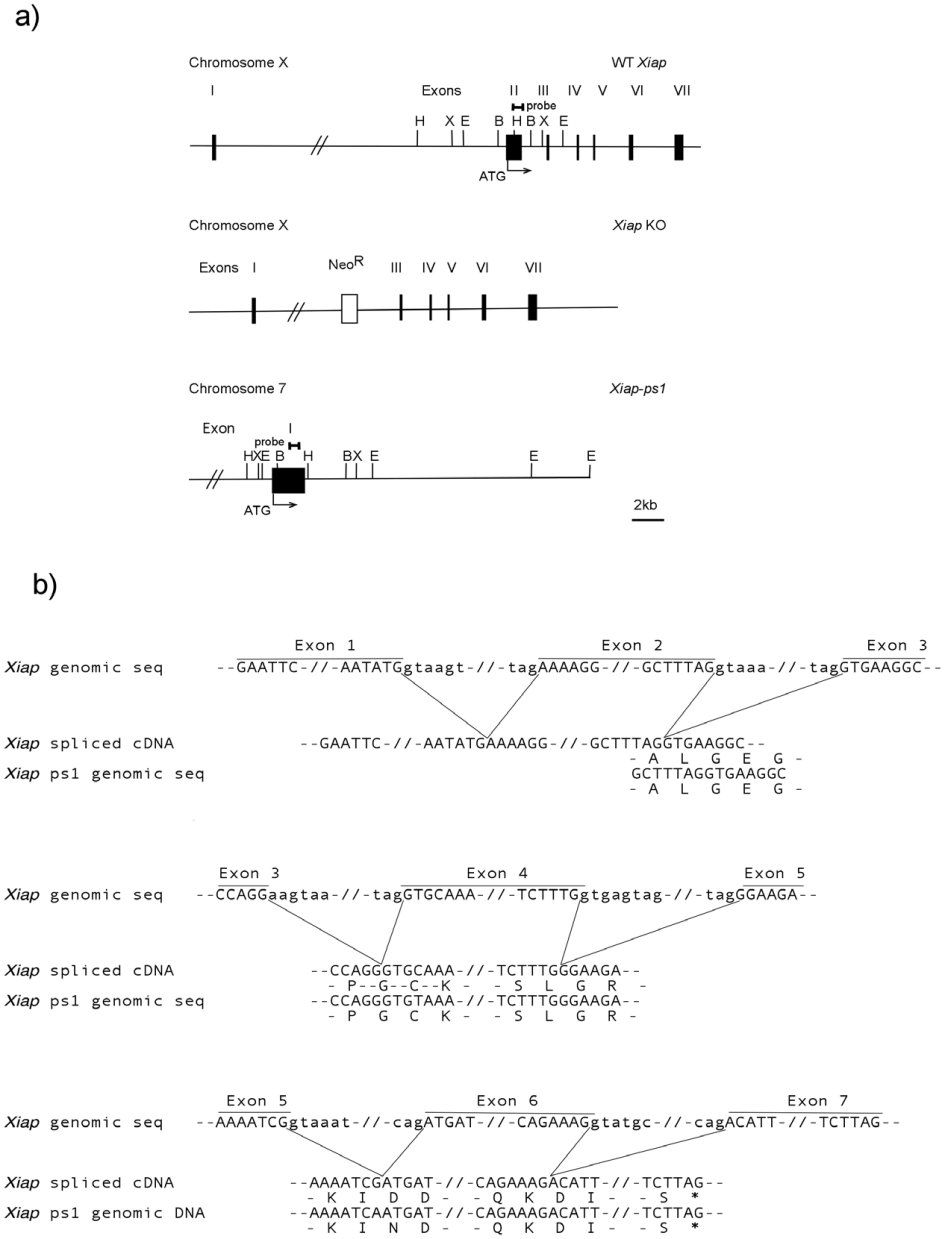
Schematic diagram of the *Xiap* genes. (a) The murine *Xiap* gene spans 42 kb on the X-chromosome and consists of 7 exons. *Xiap* K/O locus has exon 2 removed via homologous recombination. The *Xiap-*like gene is found on chromosome 7 and lacks any intronic sequences, giving rise to one exon that is 92% identical to spliced WT *Xiap*. A DNA probe designed to detect this pseudogene was produced from a 510 bp region of exon 2 in WT *Xiap* that is unable to hybridise to the *Xiap* K/O locus.(b) The splicing of the 7 exons of *Xiap* gives rise to mRNA encoding the XIAP protein. Two codons from the beginning and the end of each exon were aligned to *Xiap-ps1*. The nucleotide sequences of *Xiap* and *Xiap-ps1* are identical around the exon boundaries with the exceptions of two C>T transitions, one silent at the beginning of exon 4 and another coding at the end of exon 5.

On mouse chromosome 7 band B3 location 37,599,271–37,600,785 is a sequence with 92% nucleotide identity to *Xiap* cDNA (Fig. 1a,b). To confirm the existence of the *Xiap-ps1* gene in the C57BL/6 mouse genome we performed Southern blot analysis of genomic DNA from both wild-type (WT) and *Xiap* deleted mouse embryonic fibroblasts (MEFs), as well as from tissues from C57BL/6 WT and *Xiap* deleted mice.

As indicated in Fig. 1a, digestion of WT DNA with *Bam*HI, *Eco*RI, *Hin*dIII and *Xba*I restriction enzymes is predicted to produce fragments of the mouse *Xiap* gene of 2.1 kb, 6.8 kb, 6 kb and 2 kb, respectively, that would be able to hybridise to an *Xiap* cDNA probe containing the coding region of exon II. In the *Xiap* mutant mice, exon II of *Xiap* has been deleted by homologous recombination, and therefore genomic DNA isolated from these mice does not contain any *Xiap* sequences that are capable of hybridising with the probe.

**Figure 2:**
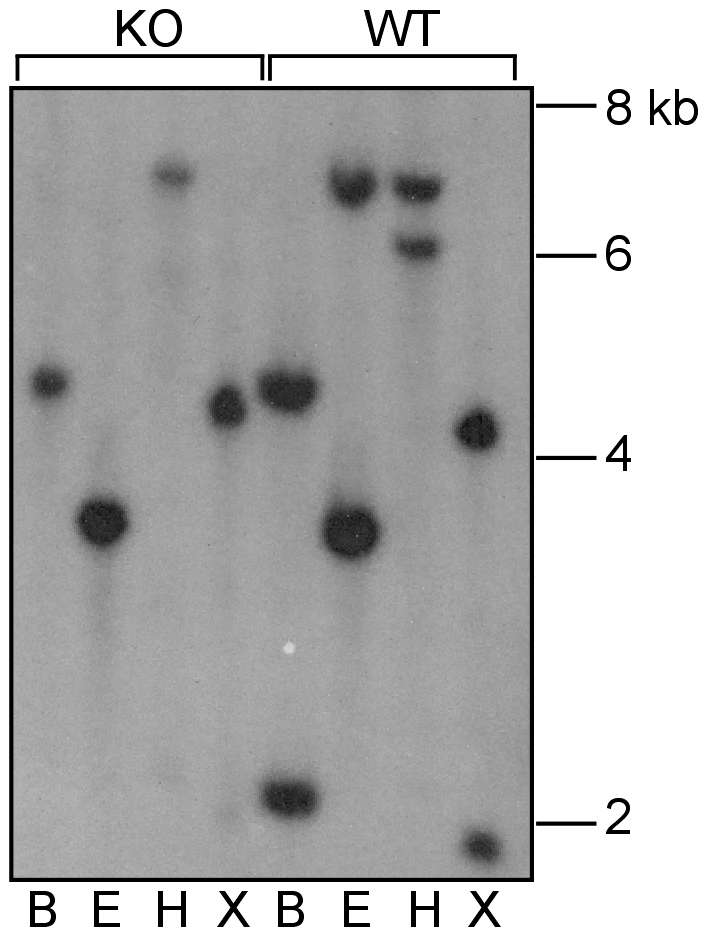
Detection of an *Xiap* pseudogene by Southern analysis. Genomic DNA was digested to completion with *Bam*HI (B), *Eco*RI (E), *Hin*dIII (H), or *Xba*I (X), and probed with a 510 bp fragment from exon 2 of *Xiap*. Bands of the expected size for the *Xiap* gene were revealed in the WT DNA. In addition, both WT and *Xiap* K/O DNA showed bands of the sizes predicted from the sequence of the *Xiap*-like gene on chromosome 7.

Any bands that hybridise at high stringency (65°C; 0.3x SSC) to the *Xiap* probe in the DNA from *Xiap* knockout mice indicate the presence of another gene similar to *Xiap*. As seen in Fig. 2, hybridisation of the probe to WT samples not only gave rise to fragments of the expected size for *Xiap*, but also additional bands that were also seen in the DNA from the *Xiap*-deleted mice. This confirms the presence of a *Xiap*-like gene that we have designated *Xiap-ps1*. These bands of 4.2 kb, 3.4 kb, 6.9 kb and 4.4 kb in samples digested with *Bam*HI, *Eco*RI, *Hin*dIII and *Xba*I, respectively, are consistent with those predicted from the digestion of the *Xiap-ps1* gene sequence on chromosome 7 in Genbank (http://apr2006.archive.ensembl.org/Mus_musculus/domainview?domainentry=IPR001370) with the same restriction enzymes (Fig. 1a).

### *Xiap-ps1* Is Not Expressed

**Figure 3:**
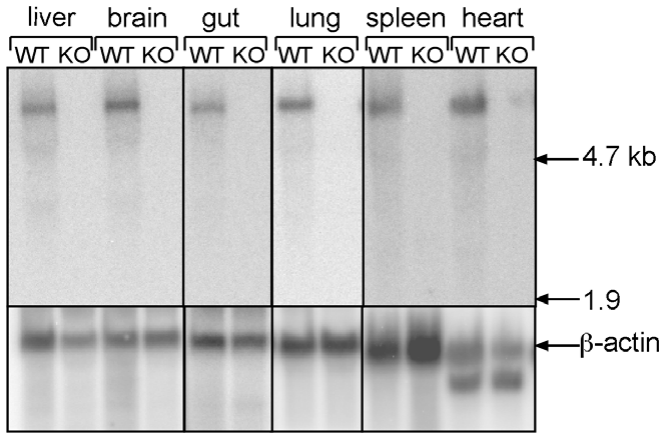
Confirmation of RNA expression by Northern analysis. Total RNA was harvested from C57BL/6 and *Xiap* K/O tissues, separated by electrophoresis, blotted and probed with a 510 bp *Xiap* fragment as indicated in Figure 1. The only band detected (∼6.6 kb) is the reported size for *Xiap* mRNA, and was only found in wild-type tissues and not in the *Xiap* knockout tissues. A β-actin probe was used to re-probe the same blots to show relative loading of lanes.

To determine whether *Xiap-ps1* is expressed we performed RNA Northern blot analyses. Tissues from WT and *Xiap* deleted mice were harvested and total RNA was isolated. The RNA samples were separated on denaturing formaldehyde agarose gels, transferred to membrane and probed with the same ^32^P-labelled probe that was used in the genomic Southern analyses. As shown in Fig. 3, in all WT tissue samples a single band of approximately 6.6 kb was detected, consistent with previous reports of the size of mouse *Xiap* mRNA [7], [9]. In contrast, in samples from *Xiap* knockout mice no bands were detected, indicating that the *Xiap-ps1* mRNA is not expressed at detectable levels in these tissues *in vivo*.

### *Xiap-ps1* Bears Premature Stop Codons

**Figure 4:**
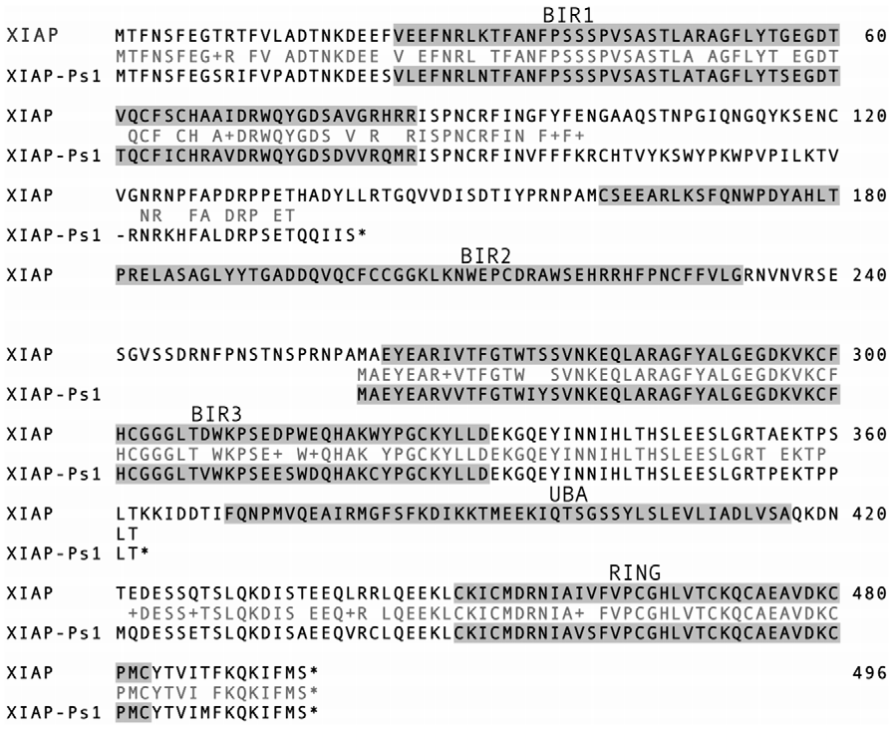
Alignment of translated pseudogene to WT XIAP protein. Three small methionine-initiated peptides are predicted from translation of the pseudogene sequence in all three frames. Functional domains are highlighted by background shading. XIAP-ps1 peptides encompassing BIR 1 and the RING domain are translations from the first frame, whereas the peptide containing BIR3 is a translation in the 3^rd^ frame. Alignment of these peptides to WT XIAP shows significant similarity with regions of XIAP, as shown in pale grey lettering, but no full length protein could be produced.

Analysis of the *Xiap-ps1* nucleotide sequence showed that it does not code for a full-length protein due to the presence of premature stop codons. Translation of the gene sequence in all three possible reading frames gives rise to several truncated polypeptide sequences (Fig. 4). This indicates that even if *Xiap-ps1* were transcribed, the presence of these premature stop codons would prevent translation of a functional IAP protein.

## Discussion

From the Southern analyses (Fig. 2) we have confirmed that a novel *Xiap*-like gene exists on mouse chromosome 7. As we detected the presence of the *Xiap-ps1* gene in genomic DNA samples from *Xiap−/−* tissues, the bands detected were not the result of the probe hybridising to the *Xiap* gene on the X chromosome. We failed to detect any evidence by Northern analysis that *Xiap-ps1* is expressed in tissues in which *Xiap* is clearly expressed at the mRNA level. Analysis of the nucleotide sequence showed that *Xiap-ps1* gene is devoid of intronic sequences found in *Xiap* and analysis of the various splicing variants showed that *Xiap-ps1* shows similarity to the regions in common to all variants, starting 7 nucleotides upstream of the initiation codon in exon 2 of *Xiap* (Fig. 1b). This suggests that *Xiap-ps1* has arisen from retrotransposition of a processed *Xiap* mRNA to chromosome 7. We hypothesise that a retroviral infection occurred in mouse germ cells that allowed insertion of reverse-transcribed spliced *Xiap* mRNA into chromosome 7. A similar event has been shown to have occurred during evolution of the great apes, leading in that case to a transcribed and translated product, BIRC8 (ILP-2), from a single-exon gene [10].

Examination of the flanking genomic sequences of *Xiap-ps1* using the Transfac database revealed several transcriptional elements, the closest to the gene being a retroviral TATA box and a CAP signal for transcription initiation 1692 and 1629 bp upstream of the initiating methionine respectively. The presence of viral elements supports our hypothesis that retrotransposition of the *Xiap* mRNA occurred.

Translation of the sequence for *Xiap-ps1* indicates that no full length protein could be produced. Although three of the larger peptide sequences were very similar to regions of XIAP, it is likely that even if these peptides were expressed within cells they would not be able to function as inhibitors of apoptosis.
